# Supplementary material for: Metabolic syndrome, social isolation, and sarcopenia in mild cognitive impairment: A multifaceted analysis of risk factors and mediating pathways
Source: PLoS One. 2025 Oct 9;20(10):e0333266. doi: 10.1371/journal.pone.0333266 (PMC12510577; doi:10.1371/journal.pone.0333266)
Supplement: S1 File — The data interpretation of the raw data mentioned in this article is located in this file. (DOCX) [file pone.0333266.s002.docx]

README

age_group：

1：＜45year

2：45year—49year

3：50year—59year

4：60year—69year

5：70year—79year

6：80year—89year

7：90year—99year

8：≥100year

Gender:

1：man

2：woman

Location：

1：Rural Village

2：Urban Community

marital_status：

1：Unmarried

2：Married

Education：

1：No formal education

2：High school and below

3：Above high school

Smoke：

1：No

2：Yes

Drink：

1：Low frequency

2：Intermediate frequency

3：High frequency
